# Aurora A plays a dual role in migration and survival of human glioblastoma cells according to the CXCL12 concentration

**DOI:** 10.1038/s41388-018-0437-3

**Published:** 2018-08-06

**Authors:** Estelle Willems, Matthias Dedobbeleer, Marina Digregorio, Arnaud Lombard, Nicolas Goffart, Paul Noel Lumapat, Jeremy Lambert, Priscilla Van den Ackerveken, Martyna Szpakowska, Andy Chevigné, Felix Scholtes, Bernard Rogister

**Affiliations:** 1 Laboratory of Nervous System Diseases and Therapy, GIGA-Neuroscience, University of Liège, Liège, Belgium; 2 Department of Neurosurgery, CHU of Liège, Liège, Belgium; 3 Department of Infection and Immunity, Immuno-Pharmacology and Interactomics, Luxembourg Institute of Health, Esch-sur-Alzette, Luxembourg; 4 Department of Neurology, CHU of Liège, Liège, Belgium

**Keywords:** Cancer stem cells, Cancer microenvironment, CNS cancer, Cell migration, Target validation

## Abstract

Primary glioblastoma is the most frequent human brain tumor in adults and is generally fatal due to tumor recurrence. We previously demonstrated that glioblastoma-initiating cells invade the subventricular zones and promote their radio-resistance in response to the local release of the CXCL12 chemokine. In this work, we show that the mitotic Aurora A kinase (AurA) is activated through the CXCL12–CXCR4 pathway in an ERK1/2-dependent manner. Moreover, the CXCL12–ERK1/2 signaling induces the expression of Ajuba, the main cofactor of AurA, which allows the auto-phosphorylation of AurA.

We show that AurA contributes to glioblastoma cell survival, radio-resistance, self-renewal, and proliferation regardless of the exogenous stimulation with CXCL12. On the other hand, AurA triggers the CXCL12-mediated migration of glioblastoma cells in vitro as well as the invasion of the subventricular zone in xenograft experiments. Moreover, AurA regulates cytoskeletal proteins (i.e., Actin and Vimentin) and favors the pro-migratory activity of the Rho-GTPase CDC42 in response to CXCL12. Altogether, these results show that AurA, a well-known kinase of the mitotic machinery, may play alternative roles in human glioblastoma according to the CXCL12 concentration.

## Introduction

Primary glioblastoma (GBM), the most common subtype of glioma (WHO grade IV), is also the most aggressive human brain tumor in adults. The standard therapy of GBM includes surgical resection, radiotherapy (RT), and usually chemotherapy with temozolomide [1, 2]. The overall survival (OS) of GBM patients does generally not exceed 15 months due to tumor recurrences, which are fatal in 90% of the cases within 5 years post-diagnosis [3, 4]. Gliomagenesis is sustained by rare self-renewing tumor cells, called GBM-initiating cells (GICs), likely involved in tumor growth and recurrence [5]. GICs share similar properties with neural stem cells (NSC) (i.e., spheroids formation and expression of stemness markers) and exhibit the unique ability to initiate tumor development in mouse models [6].

We previously demonstrated a specific tropism of GICs for the subventricular zones (SVZ), which are large neurogenic niches lining the adult lateral ventricles [7]. We showed that the SVZ-secreted CXCL12 binds the G-protein coupled receptor (GPCR) CXCR4 at the surface of GICs to promote migration, radio-resistance, and mesenchymal activation [8, 9]. CXCL12 can also bind CXCR7 (currently renamed ACKR3), an atypical GPCR unable to induce G-protein signaling and initially considered as a scavenger receptor. CXCR7 was later shown to signal through G-proteins in GBM cells, thereby opening the controversy [10, 11].

In this work, we identify the serine/threonine (ser/thr) Aurora A (AurA) mitotic kinase as a new target of the CXCL12 signaling pathway. The best-known role of AurA is to unlock G2/M transition via centrosome maturation and bipolar spindle establishment during cell division [12]. AurA overexpression in GBM promotes proliferation, survival, and therapeutic resistance of GBM cells in culture, as well as in mouse xenografts [13–16]. Increasing evidence support the role of AurA in cell migration and invasion through regulation of cytoskeleton dynamics and epithelial-to-mesenchymal transition (EMT) [17].

In the present work, we show that AurA confers a survival advantage to GBM cells regardless of the exogenous stimulation with CXCL12. On the other hand, AurA promotes GBM cell migration in response to CXCL12 stimulation and invasion of the SVZ. We therefore suggest that AurA plays a dual role in migration and survival of GBM cells according to the CXCL12 concentration. This work sheds new light on our understanding of the invasive tumor behavior that represents a relevant prognostic factor for GBM patients.

## Results

### CXCL12 activates AurA through CXCR4 and ERK1/2 proteins in glioblastoma cells

**Fig. 1:**
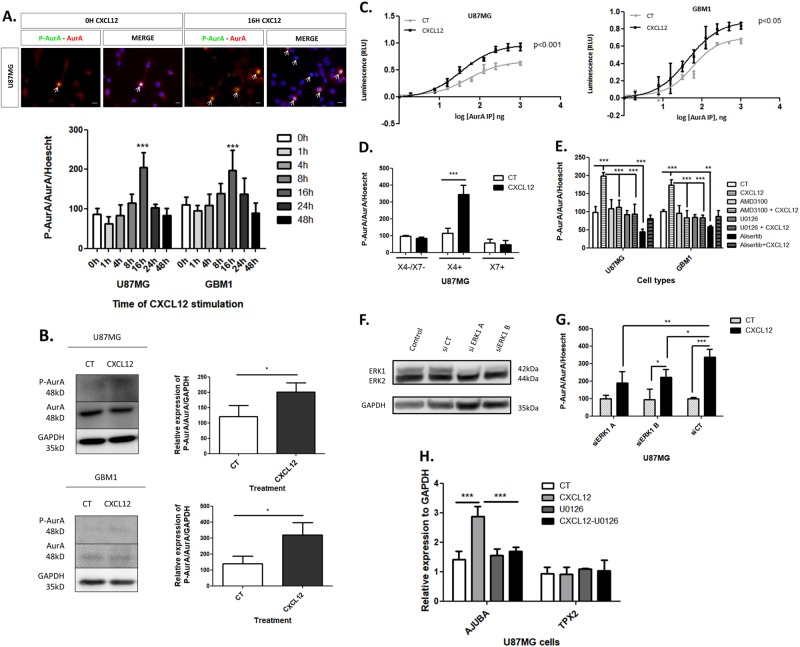
CXCL12 activates AurA through CXCR4 and ERK1/2 proteins in glioblastoma cells. **a** Immunofluorescent staining and normalized percentage of P-AurA and AurA/Hoechst double-positive U87MG and GBM1 cells after 0, 1, 4, 8, 16, 24, 48 h of CXCL12 stimulation (12.5 nM). Pictures show P-AurA (red), AurA (green), and Hoechst (blue) staining of U87MG stimulated during 16 h with 0 or 12.5 nM CXCL12. The arrows show P-AurA/AurA double-positive cells (40×). Scale bars = 10 μm, (*n* = 3). **b** Western-blot analysis of P-AurA, AurA, and GAPDH in U87MG and GBM1 cells control (CT) or stimulated during 16 h with CXCL12 (12.5 nM) (*n* = 3). **c** AurA kinase activity assay on U87MG and GBM1 cells upon 16 h of stimulation with CXCL12 (12.5 nM). Graphs show quantified luminescence (in Relative Luminescence Units, RLU) plotted semi-logarithmically against the of AurA immunoprecipitate amount (*n* = 3). **d** Normalized percentage of P-AurA and AurA/Hoechst double-positive U87MG cells immunofluorescent staining after CXCL12 stimulation (16 h, 12.5 nM) (*n* = 3). U87MG X4/X7− are negative for CXCR4 and CXCR7, U87MG X4+ overexpress CXCR4, and U87MG X7+ overexpress CXCR7. **e** Normalized percentage of P-AurA and AurA/Hoechst double-positive U87MG and GBM1 cells from immunofluorescent staining after CXCL12 stimulation (16 h, 12.5 nM). U87MG and GBM1 cells were treated with AMD3100 (24 h, 25 nM), U0126 (24 h, 10 μM), and Alisertib (48 h, 5 nM) treatments (*n* = 3). **f** Western-blot analysis of ERK1/2 and GAPDH in U87MG cells control (un-transfected) or transfected with siRNA CT (scramble control), siERK1 A, and siERK1 B. **g** Normalized percentage of P-AurA and AurA/Hoechst double-positive U87MG and GBM1 cells from immunofluorescent staining after CXCL12 stimulation (16 h, 5 nM). U87MG cells were transfected with siRNA CT (scramble control), siERK1 A, and siERK1 B (*n* = 3). **h** Quantitative RT-PCR analysis of Ajuba and TPX2 in U87MG cells after CXCL12 and/or CXCL12 treatments (*n* = 3). Graphs are mean values ± SD and are representative of three independent experiments, *p < 0.05, **p < 0.01, ***p < 0.001 (*t*-tests, 1-way ANOVA, 2-way ANOVA corrected by post-tests if appropriate)

The phosphoproteome analysis of U87MG cells by mass spectrometry reveals a significant dephosphorylation of AurA at the Thr288 residue after 1 h of CXCL12 stimulation (12.5 nM) (fold change: −12.4) (Suppl. Figure 1). To study the kinetics of AurA phosphorylation, P^Thr288^-AurA (red), AurA (green), and Hoechst (blue) positive U87MG and GBM1 cells (white arrows) were quantified by immunofluorescence after 0, 1, 4, 8, 16, 24, and 48 h of stimulation with CXCL12 (12.5 nM) (Fig. 1a). Figure 1a shows a small trend of dephosphorylation after 1 h (*p* = 0.6624, two-way ANOVA), and, surprisingly, a statistically significant AurA phosphorylation after 16 h of CXCL12 stimulation in U87MG (2.4 fold) and GBM1 (1.8 fold) cells. More precisely, the percentage of P-AurA/AurA-positive cells rises from 11.03 to 22.12% in U87MG cells and from 17.63 to 30.40% in GBM1 cells. The increased phosphorylation of AurA after CXCL12 stimulation (16 h, 12.5 nM) was also confirmed by Western blot analysis of P-AurA/AurA/GAPDH in U87MG (3.2 fold, upper panel) and GBM1 (2.4 fold, lower panel) cells (Fig. 1b).

Interestingly, the phosphorylation of AurA at the Thr288 residue is associated with an elevated AurA kinase activity [18]. We therefore performed enzymatic tests that revealed an increase in AurA kinase activity in CXCL12-stimulated GBM cells (16 h, 12.5 nM) (Fig. 1c). In Fig. 1c, we observe a reduction of EC_50_ values of the luminescence generated from the kinase reaction from 52 to 34 ng of the AurA immuno-precipitate (AurA-IP) in U87MG cells (1.5 fold, *p* < 0.001) and from 58 to 43 ng of the AurA-IP in GBM1 cells (1.3 fold, *p* < 0.05) after CXCL12 stimulation (16 h, 12.5 nM). The upper shift of luminescence suggests that CXCL12 stimulation increases the concentration of AurA activity in the immuno-precipitate. Thus, less amount of AurA-IP is required for the kinase reaction in CXCL12-stimulated U87MG and GBM1 cells.

Afterwards, we aimed to evaluate the role of CXCR4 and CXCR7 receptors, which are the two RCPG able to bind CXCL12, in the CXCL12-mediated regulation of AurA. In this context, we quantified the percentage of P-AurA/AurA/Hoechst-positive cells by immunofluorescence in (i) U87MG cells negative for CXCR4 and CXCR7 (X4−/X7−), (ii) U87MG cells overexpressing CXCR4 (X4+), or (iii) CXCR7 (X7+) (Fig. 1d). In response to CXCL12 stimulation (16 h, 12.5 nM), AurA phosphorylation is enhanced in U87MG X4+ cells (3 fold), while unaffected in U87MG X4−/X7− and X7+ cells (Fig. 1d).

Similarly, Fig. 1e shows that AMD3100 (24 h, 25 nM), a specific antagonist of CXCR4, reduces the percentage of P-AurA/AurA/Hoechst-positive U87MG (1.7 fold) and GBM1 (2.1 fold) cells in response to CXCL12 stimulation (16 h, 12.5 nM) in quantitative immunofluorescence. As a negative control, GBM cells were treated by Alisertib (48 h, 5 nM), a specific inhibitor of P ^Thr288^-AurA. Alisertib treatment decreases the percentage of P-AurA-positive U87MG and GBM1 cells regardless of the concentration of CXCL12. AurA phosphorylation is reduced (i) by 2.2 fold in U87MG cells and 1.7 fold in GBM1 cells between CT and Alisertib conditions and (ii) by 2.4 fold in U87MG and 1.9 fold in GBM1 between CXCL12 and CXCL12 + Alisertib conditions (Fig. 1e) [19].

To identify the potential regulators of CXCL12-induced AurA phosphorylation, we evaluated the role of the ERK1/2 proteins, known to be crucial for CXCL12 signaling [20]. Moreover, CXCL12-induced phosphorylation of ERK1 was identified by mass spectrometry analysis (Suppl. Figure 1) and validated by Western-blot analysis (data not shown). In Fig. 1e, AurA phosphorylation induced by CXCL12 (16 h, 12.5 nM) was quantified by immunofluorescence after ERK1/2 inhibition with U0126 (24 h, 10 μM). U0126 treatment abrogates the CXCL12-induced phosphorylation of AurA in U87MG (2.1 fold) and GBM1 (2 fold) (Fig. 1e), indicating the role of ERK1/2 in the CXCL12-AurA signaling. We also studied the specific role of ERK1 in U87MG cells transiently transfected by two different siRNA targeting ERK1 (siERK1 A and siERK1 B) or by siRNA scramble control (siCT). Western-blot analyses reveal the effective inhibition of ERK1 in siERK1-transfected cells compared to siCT-transfected and non-transfected U87MG cells (Fig. 1f). Thereafter, we assessed the impact of siERK1 transfection on CXCL12-induced phosphorylation of AurA using immunofluorescence. Figure 1g shows that siERK1 A transfection decreases the percentage of P-AurA/AurA/Hoechst-positive U87MG cells after CXCL12 stimulation. However, U87MG cells transfected with siERK1 B exhibit an increased AurA phosphorylation induced by CXCL12, potentially due to an insufficient inhibition of ERK1.

The main mode of AurA activation is Thr288 auto-phosphorylation after binding to co-factors. We therefore studied the impact of CXCL12 stimulation on the mRNA levels of Ajuba and TPX2, two crucial co-factors of AurA [21, 22]. In Fig. 1h, quantitative RT-PCR analysis shows stable TPX2/GAPDH mRNA levels, but a doubling of Ajuba/GAPDH mRNA levels in response to CXCL12 stimulation (16 h, 12.5 nM) (2 fold). In addition, Ajuba expression induced by CXCL12 is abolished by U0126 (24 h, 10 μM), suggesting that AurA phosphorylation may be mediated by Ajuba in a CXCL12–ERK1/2-dependent manner. This sequence of events explains the time course of AurA phosphorylation in response to the CXCL12 stimulation.

### Expression of CXCR4 and AurA in human glioblastoma

**Fig. 2:**
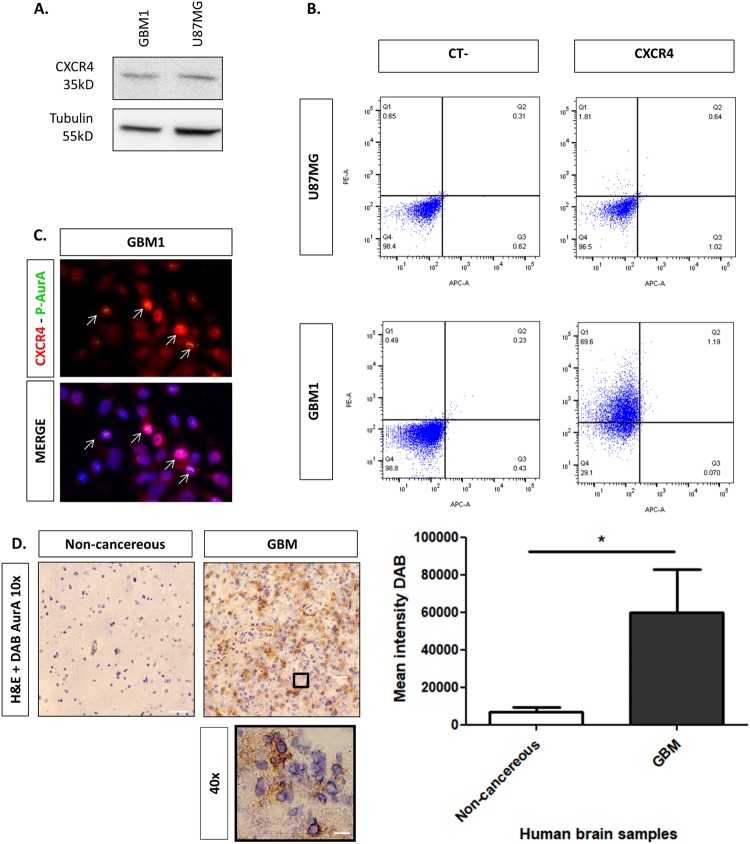
Expression of CXCR4 and AurA in human glioblastoma. **a** Western-blot analyses of CXCR4 and Tubulin in GBM1 and U87MG (*n* = 3). **b** FACS analyses of CXCR4 cell surface expression on GBM1 and U87MG cells (CT−: no antibody, CXCR4: mAb 12G5-PE) (*n* = 3). **c** Immunofluorescent stainings of CXCR4 (red), P-AurA (green), and Hoechst (blue) in GBM1 cells (*n* = 3). **d** Immunohistochemistry analysis of AurA (-DAB) in human non-cancerous (i.e., temporal lobe from epileptic patient) (*n* = 3) and GBM (*n* = 3) tissues (hematoxylin staining, 10×). The lower panel shows GBM tissue at 40× magnification. The graph shows the mean intensity of AurA staining by DAB in non-cancerous and GBM tissues quantified by Image J software. Scale bars = 100 μm. Graphs are mean values ± SD and are representative of three independent experiments, *p < 0.05, **p < 0.01, ***p < 0.001 (*t*-test corrected by post-tests if appropriate)

We then evaluated the expression and the potential clinical significance of CXCR4 and AurA in human GBM cells. Western-blot analyses show that CXCR4 is expressed both in GBM1 and U87MG cells (Fig. 2a). Flow cytometry experiments reveal that 1.16% of U87MG cells and 69.11% of GBM1 cells express CXCR4 at cell surface, suggesting that GBM1 cells may be enriched in CXCR4-overexpressing GIC compared to U87MG cells (Fig. 2b). Moreover, high CXCL12 concentration may increase the number of U87MG cells expressing CXCR4 at cell surface.

In this regard, we performed co-immunostaining of CXCR4 (red) and P-AurA (green) in U87MG cells (Fig. 2c). Figure 2c suggests that CXCR4 (red) expression predominates in the nucleus of most of GBM1 cells and decreases in cytoplasm and at cell membrane. Interestingly, P-AurA (green) is rather detected in CXCR4-highly positive GBM1 cells, reinforcing the link between AurA regulation and CXCR4 expression.

In Fig. 2d, H&E staining on resected tissue samples from GBM or epileptic patients shows a higher mean intensity of AurA staining in GBM tissues compared to non-cancerous tissues. Analysis of the Rembrandt database (*n* = 450) reveals that *CXCR4, Aurka*, and *Ajuba* mRNAs are increased in GBM (*n* = 215) compared to non-cancerous tissues (i.e., uninvolved brain tissues from GBM patients and frontal lobe of epileptic patients) (Suppl. Figure 2a) [23]. In the TCGA database, the OS of GBM patients treated with radiotherapy is lower in high-*Aurka* mRNA patients (*n* = 15) compared to low-*Aurka* mRNA patients (*n* = 24), which might hint to a prognostic value of AurA abundance in GBM patients (Suppl. Figure 2B).

### AurA and CXCL12 promote glioblastoma cell survival, radio-resistance, self-renewal, and proliferation

**Fig. 3:**
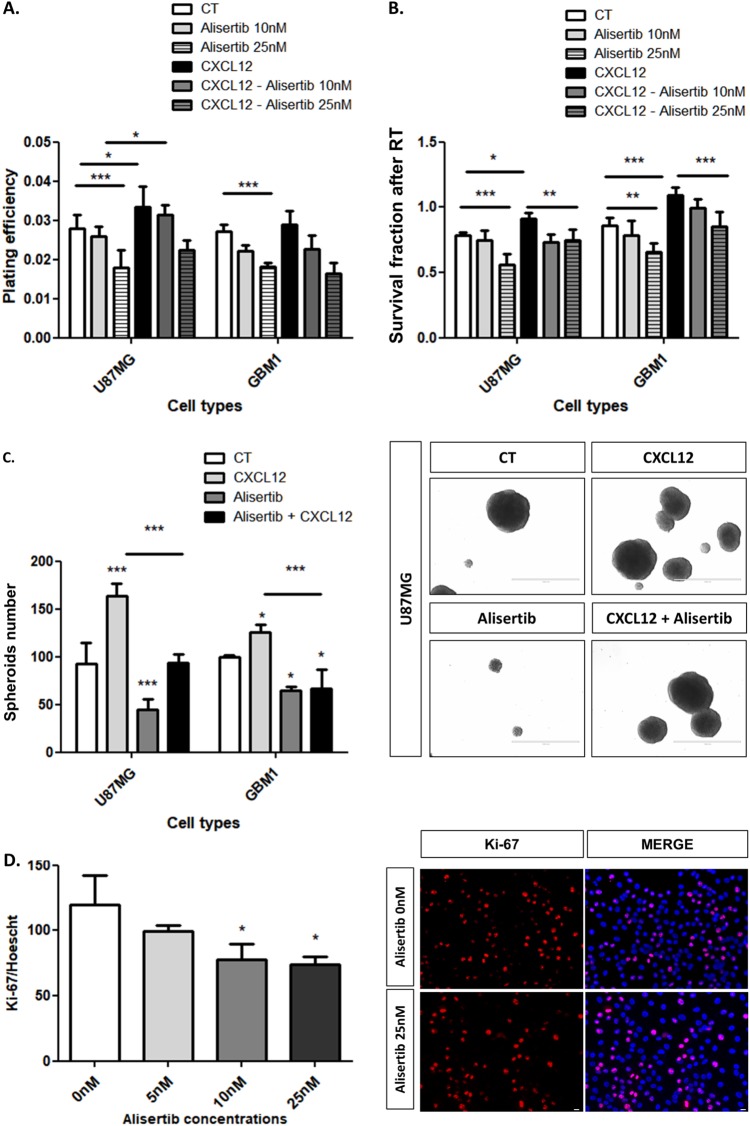
AurA and CXCL12 promote glioblastoma cell survival, radio-resistance, self-renewal, and proliferation. **a** Plating efficiency of U87MG and GBM1 cells clonogenic assays after Alisertib (48 h; 0, 10, 25 nM) and CXCL12 (16 h; 0, 100 nM) treatments (*n* = 4). **b** Survival fraction after RT of U87MG and GBM1 cells clonogenic assays after Alisertib (0, 10, 25 nM) and CXCL12 (0, 100 nM) treatments (*n* = 4). Pictures show the survival fractions of U87MG cells for each condition. Scale bars = 10 μm. **c** Quantification of spheroids of U87MG and GBM1 cells after treatment with Alisertib (25 nM) and CXCL12 (100 nM) (*n* = 3). Pictures show spheroids formation from U87MG cells for each condition. Scale bars = 50 μm. **d** Immunofluorescent staining and normalized percentage of Ki-67/Hoechst double-positive U87MG cells 48 h after 5, 10, and 25 nM Alisertib treatment. Pictures show Ki-67 (red) and Hoechst (blue) staining of U87MG cells treated with 0 or 25 nM of Alisertib (20×) Scale bars = 10 μm (*n* = 3). Graphs are mean values ± SD and are representative of three independent experiments, *p < 0.05, **p < 0.01, ***p < 0.001 (1-way ANOVA and 2-way ANOVA corrected by post-tests if appropriate)

We previously showed that SVZ-secreted CXCL12 promotes GBM cell survival after RT [8] and we, thus, tested in clonogenic assays if AurA may be involved in this process (Fig. 3a, b). In Fig. 3a, the plating efficiency is lowered after AurA inhibition with Alisertib (48 h, 25 nM) in U87MG (1.5 fold) and GBM1 (1.8 fold) cells and favored by CXCL12 stimulation (16 h, 100 nM) in U87MG cells (1.2 fold). The survival fraction after RT (5 Gy) drops after Alisertib treatment (48 h, 25 nM) (U87MG: 1.4 fold, GBM1: 1.3 fold) and augments after CXCL12 stimulation (16 h, 100 nM) (U87MG: 1.1 fold, GBM1: 1.2 fold) (Fig. 3b). Moreover, adding Alisertib to CXCL12 represses the colony number formed by U87MG (1.2 fold) and GBM1 (1.3 fold) cells (Fig. 3b). These results indicate that both AurA and CXCL12 favor GBM cell radio-resistance and that AurA inhibition is sufficient to sensitize CXCL12-stimulated GBM cells to RT.

Thereafter, we aimed to study the roles of AurA and CXCL12 in GBM spheroid formation, reflecting GBM cell self-renewal. Figure 3c indicates that GBM spheroid formation is affected by Alisertib (48 h, 10 nM) (U87MG: 2.0 fold, GBM1: 1.5 fold) and increased in response to CXCL12 stimulation (16 h, 100 nM) (U87MG: 1.7 fold, GBM1: 1.2 fold). Combined treatment of Alisertib and CXCL12 decreases the number of spheroids compared to CXCL12 stimulation alone (U87MG: 1.7 fold, GBM1: 1.9 fold) (Fig. 3c). These results suggest that AurA favors GBM cell self-renewal regardless of the exogenous stimulation with CXCL12. Moreover, AurA inhibition blocks the CXCL12-induced spheroids formation.

To evaluate the role of AurA in GBM cell proliferation, we performed Ki-67 immunofluorescent staining on U87MG cells after Alisertib treatment (0, 5, 10, 25 nM) (Fig. 3d). Figure 3d reveals a concentration-dependent decrease of Ki-67-positive U87MG cells, becoming significant after 10 and 25 nM of Alisertib treatment. Altogether, this set of data indicates that AurA modulates GBM cell survival (including after RT), self-renewal, and proliferation regardless of an exogenous CXCL12 stimulation.

### AurA triggers the migration of glioblastoma cells in response to CXCL12 stimulation

**Fig. 4:**
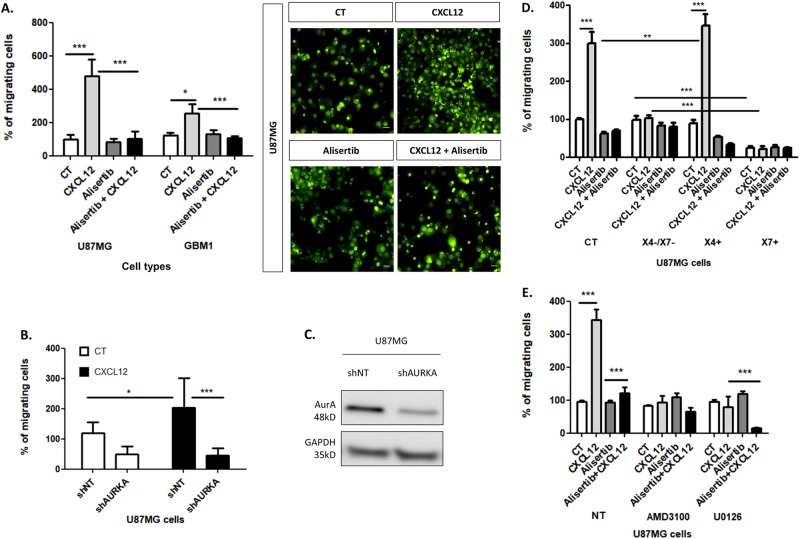
AurA promotes the migration of GBM cells in response to CXCL12 stimulation. **a** Boyden’s chambers migration assays after CXCL12 stimulation (16 h, 500 nM) of U87MG and GBM1 cells after Alisertib (48 h, 5 nM) treatments (*n* = 3). Pictures show U87MG migrating cells stained by a Cell Tracker Green for each condition (20×) Scale bars = 10 μm. **b** Boyden’s chambers migration assays after CXCL12 stimulation (16 h, 500 nM) of U87MG cells transduced shRNA directed against AurA (shAURKA) or non-targeted (shNT) (*n* = 3). **c** Western-blot analysis of AurA in U87MG cells transduced by shAURKA or shNT. **d** Boyden’s chambers migration assays after CXCL12 stimulation (16 h, 500 nM) of U87MG expressing a basal level of CXCR4 (CT, Control), negative for CXCR4 and CXCR7 (X4−/X7−), overexpressing CXCR4 (X4+) or CXCR7 (X7+) after Alisertib (48 h, 5 nM) treatment (*n* = 3). **e** Boyden’s chambers migration assays after CXCL12 stimulation (16 h, 500 nM) and Alisertib (48 h, 5 nM) treatment of U87MG cells non-treated (NT), U0126-treated (10 μM), and or AMD3100 (25 nM) (*n* = 3). Graphs are mean values ± SD and are representative of three independent experiments, *p < 0.05, **p < 0.01, ***p < 0.001 (2-way ANOVA corrected by post-tests if appropriate)

The next question was to determine if the regulation of AurA by CXCL12 could specifically affect the CXCL12-induced migration of GBM cells. Boyden chamber assays indicate that CXCL12 stimulation (16 h, 500 nM) improves the migration of U87MG cells (5.0 fold) [9] and GBM1 cells (2.5 fold) (Fig. 4). Interestingly, Fig. 4a reveals that AurA inhibition by Alisertib (48 h, 5 nM) represses GBM cell migration, exclusively in response to CXCL12 stimulation (U87MG: 6.0 fold, GBM1: 3.3 fold). The minimum required concentrations of CXCL12 and Alisertib were determined using dose–response curves (Suppl. Figure 3). To validate this result, we used U87MG transduced with *AURKA-*directed shRNA (sh*AURKA*) and non-targeted shRNA (shNT). Interestingly, the CXCL12-induced migration of U87MG^sh*AURK*A^ cells is reduced compared to U87MG^shNT^ (4.6 fold) (Fig. 4b, c). These results indicate that AurA favors GBM cell migration exclusively in response to CXCL12 stimulation.

In Fig. 4d, we evaluate the role of AurA on CXCL12-induced migration according to the expression of the CXCR4 and CXCR7 receptors, the two RCPG able to bind CXCL12 [24]. In this context, we used (i) U87MG cells naturally expressing CXCR4 and CXCR7 at cell surface (Control, CT), (ii) U87MG cells negative for CXCR4 and CXCR7 (X4−/X7−), (iii) U87MG cells overexpressing CXCR4 (X4+), or (iv) CXCR7 (X7+) (Fig. 4d). AurA inhibition by Alisertib (48 h, 5 nM) abrogates the CXCL12-induced migration of both U87MG^CT^ and U87MG^X4+^ cells (CT: 4.2 fold, X4+: 10.4 fold) (Fig. 4d). U87MG^X7+^ cells migrate less compared to control, even in the absence of exogenous CXCL12 stimulation, indicating a reduced motility of U87MG^X7+^ cells (NT: 4 fold, CXCL12: 14 fold) (Fig. 4d).

In line with these results, Fig. 4e shows that AMD3100 blocks the CXCL12-induced migration (3.7 fold) of U87MG cells. On the other hand, ERK1/2 inhibition by U0126 (24 h, 10 μM) represses U87MG cell migration (4.3 fold) in response to CXCL12 stimulation (16 h, 500 nM). Combined treatment of U0126 and Alisertib decreases the percentage of U87MG migrating cells (8 fold), potentially due to a negative feedback loop (Fig. 4e). Altogether, these data indicate the key role of the CXCL12–CXCR4–ERK1/2 signaling in AurA-mediated GBM cell migration.

### AurA modulates glioblastoma cell cytoskeleton and CDC42-dependent migration in response to CXCL12 stimulation

**Fig. 5:**
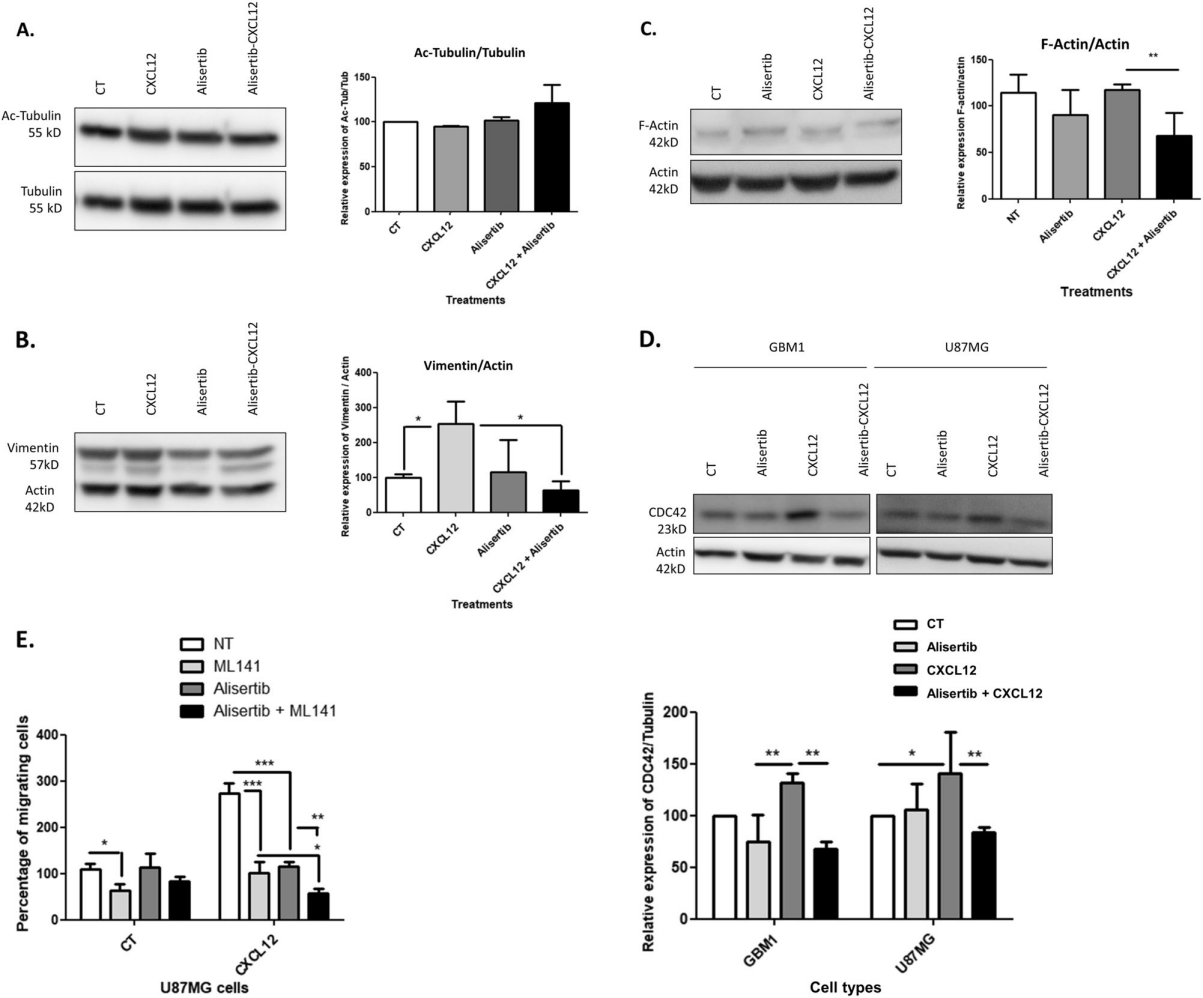
AurA modulates glioblastoma cell cytoskeleton and CDC42-dependent migration in response to CXCL12 stimulation. Western-blot analysis and relative quantification of Acetylated Tubulin/Tubulin (**a**), Vimentin/Actin (**b**), F-Actin/Actin (**c**) in U87MG cells after Alisertib treatment (48 h, 5 nM) and CXCL12 stimulation (16 h, 12.5 nM) (*n* = 3). **d** Western-blot analysis and relative quantification of CDC42/Actin in U87MG and GBM1 cells after Alisertib treatment (48 h, 5 nM) and CXCL12 stimulation (16 h, 12.5 nM) (*n* = 3). **e** Boyden’s chambers migration assays after CXCL12 stimulation (16 h, 500 nM) of U87MG and GBM1 cells after Alisertib (48 h, 5 nM) and ML141 (24 h, 10 μM) treatments (*n* = 3). Graphs are mean values ± SD and are representative of three independent experiments, *p < 0.05, **p < 0.01, ***p < 0.001 (2-way ANOVA corrected by post-tests if appropriate)

The next question was to understand how AurA influences GBM cell motility in a CXCL12-dependent way. Since AurA is reported to control tumor cell migration via cytoskeleton reorganization [25], we evaluated the role of AurA in cytoskeleton protein modification in response to CXCL12 stimulation. In this context, tubulin acetylation, actin polymerization, and vimentin expression were assessed by Western-blot analyses after Alisertib treatment of CXCL12-stimulated U87MG cells (Fig. 5a–c).

Figure 5a reveals that tubulin acetylation is unaltered in U87MG cells treated with Alisertib and/or CXCL12 in Western-blot analyses. Next, we evaluated the expression of Vimentin, the predominant intermediate filament of mesenchymal tumor cells, previously found up-regulated in CXCL12-stimulated GBM cells [26, 27]. Figure 5b indicates that Alisertib treatment represses the CXCL12-induced expression of Vimentin (4 fold) in Western-blot analyses. Finally, Fig. 5c shows that Alisertib treatment reduces the ratio of filamentous-actin (F-Actin/Actin), correlated to actin polymerization, in CXCL12-stimulated U87MG cells (1.8 fold). In conclusion, we showed that AurA contributes to the CXCL12-mediated regulation of actin polymerization and Vimentin expression in GBM cells.

We then aimed to understand how AurA may regulate actin and vimentin dynamic in a CXCL12-dependent way. In this context, we studied the regulation of the Rho GTPases signaling, which directs small Rho GTPases to the leading edge to reorganize the cytoskeleton of migrating cells [28, 29]. Western-blot analyses indicate that upstream RhoGTPase kinases, i.e., PKC and FAK, are not affected by Alisertib and CXCL12 treatments (Suppl. Figure 4) [30]. In contrast, the expression of the RhoGTPase CDC42, able to regulate cytoskeleton dynamic [29], is induced by CXCL12 stimulation (16 h, 12.5 nM) in U87MG (1.4 fold) and GBM1 (1.3 fold) cells (Fig. 5d). Furthermore, Alisertib treatment (48 h, 5 nM) abrogates the expression of CDC42 in CXCL12-stimulated GBM cells (U87MG: 1.9 fold, GBM1: 1.7 fold) (Fig. 5d).

To evaluate the functional significance of CDC42 on GBM cell migration, we performed Boyden chambers assays after CDC42 inhibition by the ML141 inhibitor (Fig. 5e). Figure 5e indicates that ML141 (24 h, 10 μM) reduces the migration of non-stimulated U87MG cells (1.7 fold) and CXCL12-stimulated U87MG cells (2.7 fold) (CXCL12: 16 h, 500 nM). Interestingly, the combined treatment of ML141 (24 h, 10 μM) and Alisertib (48 h, 5 nM) reduces the CXCL12-induced migration compared to ML141 treatment (1.8 fold) or Alisertib treatment (2 fold) alone (Fig. 5e). These data suggest that CDC42 enhances GBM cell migration whatever the CXCL12 concentration is. CDC42 and AurA could cooperate to trigger GBM cell migration in response to CXCL12 stimulation. Altogether, these results indicate that AurA up-regulates CDC42 in a CXCL12-dependent manner, which may result in cytoskeleton reorganization required for GBM cell migration [31].

### Alisertib treatment decreases the number of glioblastoma cells invading the subventricular zone in glioblastoma-xenografted mice

**Fig. 6:**
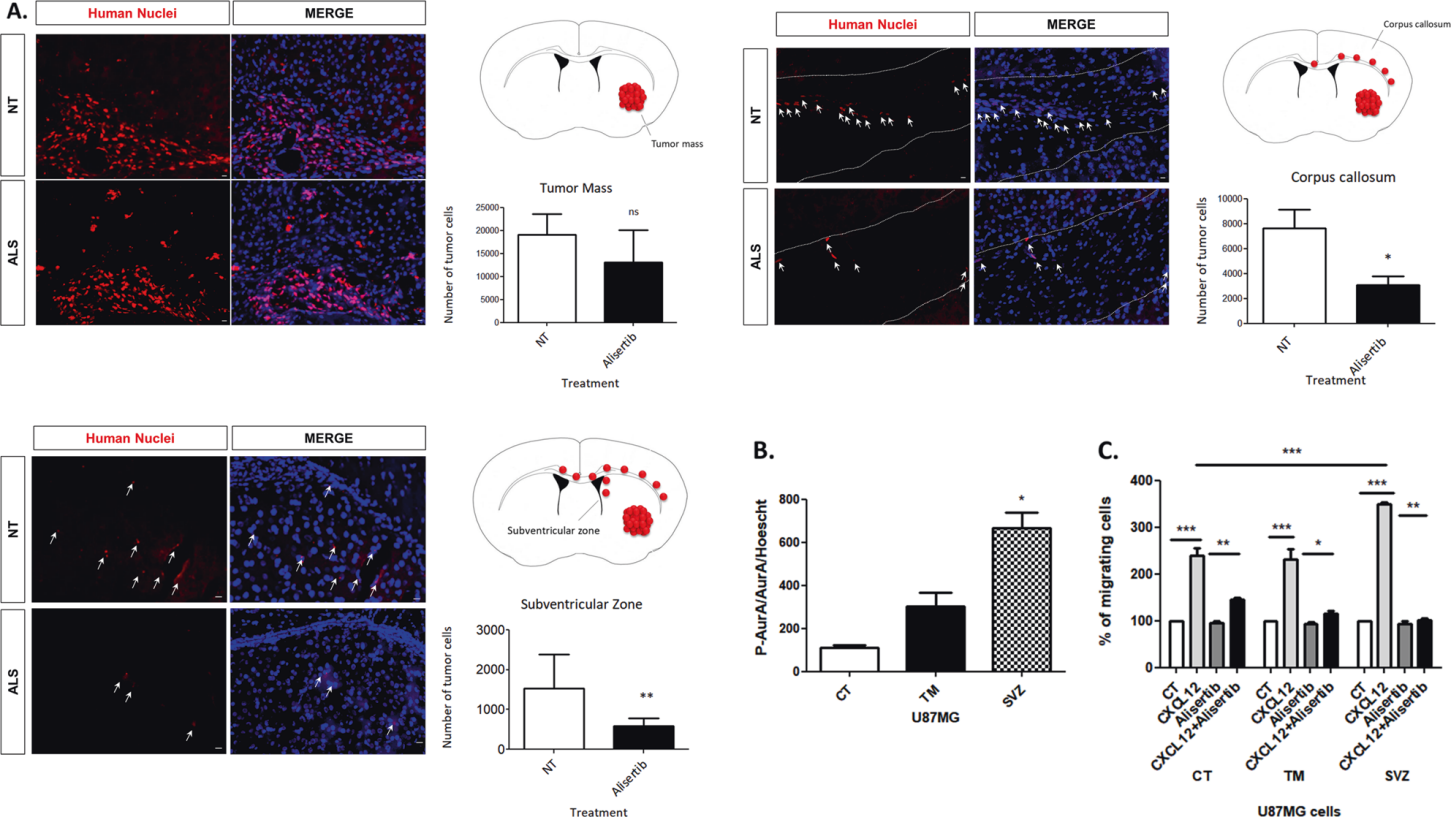
Alisertib decreases the number of glioblastoma cells invading the *corpus callosum* and the subventricular zone in glioblastoma-xenografted mice. **a** Immunofluorescent staining and normalized percentage of human nuclei (red)/Hoechst (blue) positive U87MG cells (20×) in the TM, CC, and SVZ after xenotransplantation in mice untreated (NT) (*n* = 6) or treated with Alisertib (20 mg/kg/day) (*n* = 8). **b** Immunofluorescent staining and normalized percentage of P-AurA and AurA/Hoechst (20×) double-positive U87MG cells non-grafted (CT), extracted from the TM (TM) and from the SVZ (SVZ) of U87MG-xenografted mice. **c** Boyden’s chambers migration assays of U87MG cells non-grafted (CT), extracted from the TM (TM) and from the SVZ (SVZ) of GBM-xenografted mice after Alisertib treatment (48 h, 5 nM) in response to CXCL12 (16 h, 500 nM). Graphs are mean values ± SD and are representative of three independent experiments, *p < 0.05, **p < 0.01, ***p < 0.001 (unpaired *t* test and 2-way ANOVA corrected by post-tests if appropriate)

Our next purpose was to evaluate if AurA was required for GBM cell invasion toward the SVZ, previously shown to be mediated by CXCL12 [9]. In Fig. 6, we study the impact of Alisertib treatment after U87MG intra-striatal xenotransplantation in nude immuno-deficient mice. We previously showed that U87MG cells firstly proliferate in the mouse striatum to constitute the tumor mass (TM) before starting to migrate at day 21 along the *corpus callosum* (CC) and toward the SVZ [7, 9]. Alisertib treatment was therefore performed during the fourth week after the intra-striatal graft to study the role of AurA in GBM invasion rather than tumor growth. Alisertib treatment (20 mg/kg/day) and control solution were orally administrated to two homogeneous groups of GBM-xenografted mice from day 21 to day 26 (Alisertib: *n* = 6, CT: *n* = 8). After the euthanasia of mice, U87MG cells were counted in the TM, the CC, and the SVZ via immunofluorescent staining directed against human nuclei (Fig. 6a).

Figure 6a shows representative immunofluorescences (left panels) and quantification graphs (right panels) of Human nuclei (red)/Hoechst (blue) staining in the TM, the CC, and the SVZ (20×) of GBM-xenografted mice. No significant change is observed in the number of U87MG cells constituting the TM (Fig. 6a, upper panel). In contrast, the numbers of U87MG cells found in the CC (2.35 fold) and in the SVZ (2.30 fold) are reduced in Alisertib-treated animals compared to control group (Fig. 6a, middle and lower panels). This observation suggests that AurA inhibition decreases the numbers of GBM cells invading the CC and the SVZ in GBM-xenografted mice.

In order to study the role of AurA in GBM cells invading the SVZ, we used U87MG cells extracted from the TM (U87MG TM) and the SVZ (U87MG SVZ) of GBM xenografts after establishment in culture. U87MG SVZ cells were previously described as a GIC-enriched population, characterized by their higher ability to initiate GBM tumors in mice, form spheroids and express stem cell markers [7]. In this work, we validate that the U87MG SVZ population forms more spheroids than their counterparts (i.e., U87MG TM cells) (Suppl. Figure 5A). Moreover, immunofluorescent experiments show that P-AurA (red) staining is rather found in Sox2 (green)-positive U87MG SVZ than U87MG TM cells (Suppl. Figure 5B). We then quantified the percentage of P-AurA/AurA/Hoechst-positive U87MG CT (control, non-grafted), TM and SVZ cells in immunofluorescent experiments. Figure 6b shows that AurA phosphorylation is elevated in U87MG SVZ cells compared to U87MG TM and CT cells, suggesting that GICs-enriched GBM cells extracted from the SVZ exhibit a higher AurA activity.

In Fig. 6c, we compared the pro-migratory role of AurA in U87MG CT (non-grafted), TM, and SVZ cells in Boyden chambers assays. U87MG CT, TM, and SVZ cells migrate in response to CXCL12 stimulation. Moreover, we observe that Alisertib treatment inhibits the CXCL12-induced migration of U87MG CT, TM, and U87MG SVZ cells (Fig. 6c). Interestingly, CXCL12-stimulated U87MG SVZ cells migrate significantly more than CXCL12-stimulated U87MG CT cells (i.e., non-grafted cells). On the other hand, the percentage of migration in response to CXCL12 was similar between U87MG TM and U87MG CT cells (i.e., non-grafted). Altogether, these results show that AurA inhibition is sufficient to antagonize the migratory abilities of GICs-enriched GBM cells invading the SVZ in vitro.

## Discussion

Increasing studies suggest that GICs evolve from neural progenitors and hierarchically direct gliomagenesis [32]. Clinical studies showed that human GBM tumors in contact with the SVZ, a large brain stem cell niche, are associated with shorter survival, radio-resistance, and increased risk of multifocal and distant recurrence [33, 34]. Furthermore, we previously demonstrated that GICs specifically invade the mouse SVZ in response to the local production of CXCL12 [7, 9]. The analysis of the phosphoproteome of CXCL12-stimulated U87MG cells identified, among others, the AurA mitotic kinase as a new target of CXCL12. In contrast, the phosphorylation of the other members of the Aurora kinase family (i.e., AurB and AurC) was not significantly affected. The best-known role of AurA is to trigger mitotic entry via microtubule dynamic and centrosome maturation [35]. In GBM, AurA overexpression is associated with mitotic failure, supernumerary centrosomes, proliferation, survival, and therapeutic resistance [13, 14, 36, 37]. Recent studies demonstrated that AurA also possesses oncogenic properties unrelated to cell division, which include cell polarity, EMT, migration, and invasion (reviewed in [17]).

Interestingly, we showed by immunohistochemistry that AurA is overexpressed in human GBM tissues compared to non-cancerous tissues (Fig. 2d). Similarly, bio-informatics databases revealed that *AurA, Ajuba*, and *CXCR4* mRNA levels are elevated in GBM tissues compared to non-cancerous tissues (Suppl. Figure 2). Although AurA and CXCR4 are also increased in GBM tumors compared to astrocytomas and oligodendrogliomas, the correlation between AurA/CXCR4 expression and the grade of glioma malignancy remain to be proved. Indeed, the Rembrandt database does not distinguish (i) IDH-mutant (secondary) and IDH wild-type (primary) GBM, neither (ii) grade I, II, nor III astrocytomas, which highly differ from their malignancy grade. Moreover, CXCR4 is also up-regulated by signaling pathways induced by non-tumor immune or inflammatory cells surrounding gliomas, which may mislead the interpretation of its clinical significance. Similarly, the TCGA database showed that AurA mRNA levels are correlated with poor survival of GBM patient treated with radiotherapy. However, the value of AurA as an independent prognosis factor (according to tumor size, location, surgery, and IDH status) should be determined to validate the clinical significance of AurA in human GBM.

In this work, the phosphorylation and the kinase activity of AurA were augmented in CXCL12-stimulated GBM cells, probably via CXCR4–ERK1/2–Ajuba signaling (Fig. 1). Interestingly, we observed that 10–20% of GBM cells respond to the CXCL12 stimulation by an increased AurA phosphorylation. Moreover, the phosphorylated form of AurA and the stem cell marker CD133 were rather found in CXCR4-positive GBM cells. We therefore suggest that AurA regulation by CXCL12 may depend on the expression levels of CXCR4, reported to be up-regulated in GICs (Fig. 2c) [38]. In this context, AurA phosphorylation was higher in U87MG SVZ, known to be enriched in GICs, compared to U87MG TM cells (Fig. 6b). Indeed, we previously showed that U87MG SVZ cells express stem cell markers, form an increased number of spheroids in cell culture and give rise to more GBM tumors in xenografted mice in limited cell dilution assays [7]. We therefore suggest that the GIC-enriched SVZ cell population exhibits a high AurA activity, especially in response to CXCL12 stimulation.

The next purpose was to study the role of AurA in CXCL12-stimulated GBM human cells. We showed that AurA and CXCL12 favored GBM survival, including after RT (Fig. 3b). Although AurA mediates GBM radio-resistance in the absence of exogenous CXCL12 stimulation, it is important to keep in mind that GBM cells themselves secrete CXCL12 to trigger CXCL12–CXCR4 autocrine/paracrine signaling [39, 40]. AurA-mediated GBM radio-resistance could therefore be dependent of the CXCL12 signaling pathway. It would therefore be interesting to treat GBM cells with AMD3100 and Alisertib in clonogenic assays to evaluate the interplay between AurA and CXCL12 signaling in GBM radio-resistance.

On the other hand, the role of AurA in GBM radio-resistance has been supported by previous studies, which showed that Alisertib sensitizes GBM cells to RT [13]. In this context, it would be interesting to evaluate molecular mechanisms underlying the AurA-mediated radio-resistance. AurA is reported to control DNA repair responses to RT in various cancer cells and should be investigated in GBM [41]. Given the key role of AurA in GBM cell division [15, 16, 42], AurA may potentially redistribute and arrest irradiated GBM cells in a more resistant phase of cell cycle (i.e., G0 and S-freezing) [43]. Moreover, irradiation of GBM-xenografted mice would also help to understand the role of AurA in GBM radio-resistance and in the SVZ.

In parallel to cell survival, CXCL12 also favors GBM cell migration and invasion of the SVZ [9]. Interestingly, we showed here that AurA regulated GBM cell cytoskeleton (Fig. 5) and migration specifically in response to CXCL12 (Fig. 4), as well as invasion of the SVZ in GBM-xenografted mice (Fig. 6). Furthermore, our results suggest that CXCR4 is required for CXCL12-induced migration, while U87MG X7+ cells migrate less than U87MG X4−/X7− cells (Fig. 4d). Since GBM cells endogenously secrete CXCL12, CXCR7 may counteract the CXCR4-dependent migration of GBM cells. This hypothesis is in agreement with the reported agonist activity of AMD3100, the CXCR4 inhibitor, on CXCR7 in GBM cells [44].

In conclusion, this work demonstrates that non-mitotic roles can be acquired by AurA, especially when overexpressed in cancer cells and exposed to specific extracellular stimuli. The functional plasticity of AurA, as well as its key role in the CXCL12 pathway, makes it an attractive therapeutic target in human GBM.

## Materials and methods

### Cell culture and chemicals

Human U87MG (ATCC) and GBM1 cells were cultivated in DMEM containing 10% of fetal bovine serum (FBS) (Invitrogen®, Merelbeke, Belgium) and validated as previously described [7]. Mycoplasma contamination was checked every 2 months. GBM1 cells were seeded in culture from a human GBM tumor resected at the Neurosurgical Department (CHU of Liège, Liège, Belgium) in 2011. In collaboration with the Anatomo-pathological of the GIGA Institute (Liège, Belgium), we characterized GBM1 cells according to (i) the status of p53 using immunohistochemistry staining and (ii) IDH1/IDH2 mutation and MGMT promoter methylation using PCR analyses. GBM1 cells were described as p53 wild-type cells, negative for p.R132H/p.R132C mutations for IDH1 and p.R172K/p.R172M for IDH2 and methylated at the MGMT promoter.

U87MG cells were extracted from the tumor mass (U87MG TM cells) and from the SVZ (U87MG SVZ cells) of GBM-xenografted mice as previously described [7]. Briefly, U87MG TM and SVZ were isolated from 400 μm thickness sections obtained with a Leica vibratome (Leica VT1000S, Groot Bijgaarden, Belgium). Brain slices were incubated in papain (WothingtonV R, Lakewood, NJ, USA) for 30 min at 37 °C, suspended in ovomucoide (WothingtonV R) to stop the dissociation and established in culture.

For cell culture experiments, GBM cells were treated with CXCL12 (Peprotech®, London, UK), Alisertib (MLN8237, Selleckchem®, Huissen, The Netherlands), mitomycin C (30 μM) (Sigma®, Bornem, Belgium), AMD3100 (25 nM) (Sigma®), U0126 (10 μM) (Cell Signaling Technology®, Leiden, The Netherlands), and ML141 (10 μM) (Merck Millipore®, Brussels, Belgium). The concentrations of CXCL12 and Alisertib were adapted according to the experimental requirements (see below).

### Animals and intra-striatal graft

P40 female immuno-deficient mice (Crl:NU-Foxn1nu) were obtained from Charles River Laboratories (Charles River Laboratories®, Wilmington, UK) and handled according to the Animal Ethical Committee of the University of Liège. All animals were cared for in accordance with the Declaration of Helsinki, the guidelines of the Belgium Ministry of Agriculture in agreement with the European Commission Laboratory Animal Care and Use Regulation (86/609/CEE, CE of J nL358, 18 December 1986).

Intra-striatal grafts were performed following the same previously described procedures [7]. Luciferase-carrying plasmid transduction of U87MG was used to evaluate tumor growth and establish homogeneous groups of GBM-xenografted mice before treatment. Alisertib (Selleckchem®) was formulated in 10% 2-hydroxypropyl-β-cyclodextrin/1% sodium bicarbonate (10% HBC/1% NaHCO_3_) and orally administrated at 20 mg/kg/day from day 21 to day 26 post-graft (*n* = 8 for Alisertib-treated mice and *n* = 6 for control mice). The sample size was determined by two-tail *t*-tests using the G-Power software based on previous experiments by considering a power of 95%, a risk of α error of 5% and an effect size of 2. Exclusion criteria were pre-established as follows: (i) a significant weight loss of 20% of the total body mass measured weekly from the beginning of the experiment, (ii) a significant intracranial hypertension, resulting in decreased activity and high respiratory rate. Mice were euthanized at day 28 and processed for immunofluorescence analysis. The number of cells was counted in each zone (TM, CC, SVZ) on three slides per mice and extrapolated to the total number of slides using the Image J software. This quantification method allowed us to homogenize the number of cells within and among the different zones. Tumor cells quantification was randomly performed using double-blind procedures.

### Lentiviral vectors transduction

U87MG cells transduced by lentiviral vectors were obtained in collaboration with the Lentiviral Vectors Platform of the GIGA Institute (Liège, Belgium). The U87MG^eIL^ cells, expressing EGFP and Luciferase, were obtained as previously described [9]. For U87MG^shAURKA^, U87MG were transduced with the following lentiviral vectors: GVV_353 pLV H1/TO *AURKA*1_ 655 shRNA plasmid (Cyagen®, VB151023-10056) and *eGFP* shRNA plasmid (Sigma®, SHC005) and selected using 1 mg/ml Puromicyn (Sigma®).

### Plasmid transfection

To obtain the stable U87MG^X4+^ and U87MG^X7+^ cell lines, U87MG cells (from the National Institute of Health, Luxembourg) were transfected using Lipofectamine (Life Technologies®, Merelbeke, Belgium) with a pBABE-puro vector (Addgene®, Teddington, UK) containing CXCR7 (UniProt: P25106) or CXCR4 (UniProt: P61073) sequences optimized for mammalian expression. Stable U87MG^CXCR7^ and U87MG^CXCR4^ cell lines were established following Puromicyn selection (1 or 0.5 μg/ml, respectively) and subsequent single-cell sorting using mAbs specific for CXCR7 (clone 11G8) (R&D Systems®, Abingdon, UK) or CXCR4 (clone 12G5) (BD Biosciences®, Erembodegem, Belgium) [45].

To obtain the transiently transfected U87MG^siERK1 A^ and U87MG^siERK1 B^ cell lines, U87MG cells were transiently transfected by siRNA ERK1 A (SI00606004 Qiagen®, Venlo, The Netherlands) and siRNA ERK1 B (SI00605997, Qiagen®) from FlexiTube GeneSolution (GS5595) according to the manufacturer’s instructions. Briefly, Xfect RNA Transfection Polymer was added to 25 pmol (for 24-well plates) or 100 pmol (for 6-well plates) of siRNA ERK1. After 10 min of incubation at RT, the siRNA-Xfect RNA Transfection Polymer was incubated in complete culture medium for 4 h before medium replacement. Western-blot and immunofluorescence experiments were performed 48 h post-transfection.

### Phosphoproteome analysis

U87MG cells were stimulated during 1 h with 0 or 12.5 nM human recombinant CXCL12 (*n* = 3), collected and analyzed by the Post-Translational Modifications (PTM) Scan Direct for phosphorylation: Multipathway Reagent V2.0 (Cell Signaling Technology®, Leiden, The Netherlands). The significant relative fold change was established at ±2.5 (*p* value < 0.05).

### Immunofluorescence

Immunofluorescence was performed as previously described [9]. The primary antibodies were: anti-Aurora A/AIK (rabbit monoclonal, 1:100, Cell Signaling Technology®), anti-Phospho-Aurora A (Thr288) (rabbit monoclonal, 1:100, Cell Signaling Technology®), anti-CXCR4 (mouse monoclonal, 1:100, Santa Cruz®, Heidelberg, Germany), anti-Ki-67 (mouse monoclonal, 1:400, BD Biosciences®, Erembodegem Belgium), anti-Human Nuclei (mouse monoclonal IgG, 1:250, Merck Millipore®). The specificity of the used primary antibodies was verified using IgG isotype controls for anti-Aurora A/AIK and anti-Phospho-Aurora A (Thr288) antibodies and secondary antibody incubation alone for other antibodies. The quantification of P-AurA-positive cells was normalized according to AurA/Hoechst double-positive cells per triplicated well for each condition using the Image J software (*n* = 3).

### Human tissue samples and immunohistochemistry

Human tissue samples and immunohistochemistry staining were provided by the Bio-bank and the Histology platforms of the GIGA Institute. Frozen tissues samples were fixed with acetone (15 min at 4 °C) and blocked with H_2_O_2_ 3% (15 min). The primary Anti-Aurora A antibody (rabbit polyclonal 1:500, ab1287, Abcam) was incubated 1 h at RT and revealed with DAB. Nuclei were colored with hematoxylin. Mean intensity of AurA staining by DAB was quantified using Image J software (*n* = 3 per condition).

### Western blot

Western-blots were performed as previously described [8]. The primary antibodies were: anti-Aurora A/AIK (rabbit monoclonal, 1:100, Cell Signaling Technology®), anti-Phospho-Aurora A (Thr288) (C39D8) (rabbit monoclonal, Cell Signaling Technology®), anti-GAPDH (6C5) (mouse monoclonal, Merck Millipore, Overijse, Belgium), anti-CXCR4 (C-20) (goat polyclonal 1:100, Santa Cruz®), anti-Vimentin (rabbit monoclonal 1:1000, Cell Signaling®), anti-F-actin (NH3) (mouse monoclonal, 1:500, Abcam®), anti-β-actin-peroxidase (mouse monoclonal, 1:10 000, Sigma®), anti-acetylated tubulin (mouse monoclonal 1:100, Santa Cruz®), anti-α-tubulin (mouse monoclonal 1:100, Santa Cruz®), anti-CDC42 (mouse monoclonal 1:100, Santa Cruz®, TX, USA). Western blot quantification was performed using the Image J software (*n* = 3).

### Kinase assay

Cells were treated with 0 or 12.5 nM of CXCL12 during 16 h. Protein extracts were collected 30 min after incubation in a lysis solution (10 mM Tris/Cl pH 7.5, 150 mM NaCl, 0.5 mM EDTA, 0.5% NP40). Pierce Protein A/G Magnetic Beads (Thermo Fisher Scientific®) were coated with the anti-AurA antibody (Anti-Aurora A antibody [35C1], Abcam®). Protein extracts were then incubated for 1 h at 4 °C with the AurA antibody coated A/G Magnetic Beads. Aurora A immuno-precipitation (AurA-IP) was eluted using an elution solution (200 mM glycine pH 2.5) and the supernatant was used for the kinase assay using the ADP-Glo™ Kinase Assay kit (Promega®).

The ATP to ADP standard curve was prepared from 100 μM ATP/100 μM ADP to estimate the amount of ADP produced in the kinase reaction. The Recombinant Aurora A (AurA-r) Kinase Enzyme System was used as positive control. AurA-IP and AurA-r were diluted from 2:2 in 1× kinase buffer D (Kinase Dilution Preparation) and 100 μM ATP 10×, 4× kinase Buffer D and MBP 1 mg/ml substrate were diluted in water (ATP/Substrate Preparation). The kinase reaction was performed for 1 h at RT by loading 3 μl Kinase Dilution Preparation and 2 μl ATP/Substrate Preparation in 384-well plates. Relative Luminescence Units (RLU) produced by the ATP-luciferase system was measured with a micro-plate luminometer (Thermo Fisher Scientific®) (*n* = 3).

### Quantitative RT-PCR

Eight hundred nanograms of total RNA were treated with DNAse (Promega®) and retro-transcribed by M-MLV Reverse Transcriptase (Promega®). The cDNA product was used for quantitative real-time (RT) PCR in a SYBR-Green Master Mix (Roche®) using the LightCycler 480 (Roche®). Primer (Integrated DNA Technologies®, Leuven, Belgium) sequences were designed as follows: human *AJUBA*: FOR: 5′-GGT ACC AGG ACG AGC TA-3′, REV: 5′ ATA CAG GTG CCG AAG TAG TCC-3′; human *TPX2*: FOR: 5′-GCC TTT CAA CCT GTC CCA AG-3′, REV: 5′-AGG GGT TCG TTT ATG GAA GTC T-3′. Gene expression levels were normalized according to the human GAPDH housekeeping gene (FOR: 5′-GGA CTC ATG ACC ACA GTC CAT-3′, REV: 5′-GTT CAG CTC AGG GAT GAC CTT-3′) (*n* = 3).

### Flow cytometry

CXCR4 expression at cell surface of U87MG and GBM1 cells was evaluated by flow cytometry using a phycoerythrin-conjugated mAb specific to CXCR4 (clone 12G5, 1:20; BD Biosciences) on a BD FACS Fortessa cytometer (BD Biosciences) and quantified using FlowJo v10 software as previously described (PMID 27238288).

### Clinical databases

Clinical data and mRNA levels data were obtained from the Repository of Molecular Brain Neoplasia Data (Rembrandt, http://www.betastasis.com/glioma/rembrandt/) and The Cancer Genome Atlas (TGCA) Portal (https://tcga-data.nci.nih.gov/tcga/) (See Supplementary Material for details).

From the Rembrandt database (*n* = 446), *Aurka* mRNA levels (from Affymetrix HG U133 v2.0 Plus) were obtained from GBM (*n* = 214), astrocytomas (*n* = 145), oligodendrogliomas (*n* = 66), and non-cancerous (*n* = 21) tissues. Non-cancerous tissues include uninvolved brain tissues from GBM patients and frontal lobe tissues from epileptic patients. *Aurka, CXCR4*, *and Ajuba* expression levels were analyzed by two-way ANOVA and Bartlett’s post-tests.

From the TGCA database, a cohort of 438 primary GBM patients with complete data was extracted. *Aurka* mRNA levels were extracted from Agilent G4502A level 3 data. mRNA levels were centered on the mean *Aurka* expression level. K-means clustering was used to partition patients into two clusters (*z*-score > 2)—high and low mRNA levels—regarding *Aurka* mRNA levels. Survival differences between expression groups were assessed by Kaplan–Meier curves and log-rank tests.

### Clonogenic assay

Cells were plated at a density of 500 cells per well in 6 wells-plates (Corning®), treated with 0, 10, and 25 nM Alisertib during 48 h and stimulated during 16 h with 100 nM CXCL12. 2 h after irradiation by the Gammacell® 40 Extractor with 0 or 5 Gy (Best Theratronics®, Ottawa, Canada), medium was replaced with DMEM supplemented with 10% serum. Cell colonies (10 ± 1 cells) were counted by optical microscopy 7 days after irradiation (*n* = 4). The plating efficiency (PE) was calculated as follows: PE = number of grown colonies divided by the number of seeded cells (i.e., 500 cells per well). The survival fraction after irradiation of 5 Gy (SF5) was calculated as follows: plating efficiency at 5 Gy divided per the plating efficiency at 0 Gy of the corresponding control.

### Spheroid formation assay

T25 plates (Corning®, Lasne, Belgium) were coated with 0.3 mg/ml poly-(2-hydroxyethyl methacrylate) (Sigma®) to inhibit cell adherence. Cells were seeded at a density of 25,000 cells per milliliter in a DMEM/F12 medium (Lonza®, Verviers, Belgium) containing B27 without vitamin A (Thermo Fischer Scientific®, Merelbeke, Belgium) and daily supplemented with 20 ng/ml EGF and 10 ng/ml FGF2 (Peprotech®). Spheroids were counted by optical microscopy 72 h after cell seeding and Alisertib (48 h, 25 nM) and/or CXCL12 (16 h, 100 nM) treatment(s).

### Proliferation assay

For proliferation assays, cells were seeded at a density of 50,000 cells per well in a 24-well plate. Cells were treated with 0, 5, 10, and 25 nM of Alisertib during 48 h and processed for Ki-67 immunofluorescent staining. The quantification of Ki-67-positive cells was performed by normalization of the number of Ki-67-positive cells according to the number of Hoechst-positive cells per triplicated well for each condition (*n* = 3).

### Migration assay

Migration assays were performed in 96-well chemotactic chamber with 10 μm pore at a density of 10^3^ pores per mm^2^ (Neuro Probe Inc, Gaithersburg, USA). Cells were pre-treated with Mitomycin C (16 h, 30 μM) (Sigma®), an alkylating agent used to generate mitotically inactive cells in culture. Other treatments were administrated as follows: Alisertib (48 h, 5 nM; Selleckchem®), AMD3100 (24 h, 25 nM; Sigma®), U0126 (24 h, 10 μM; Cell Signaling Technology®), ML141 (24 h, 1 μM; Merck Millipore®) (*n* = 3). Migration assays were performed as previously described [9].

Briefly, cells were stained with Cell Tracker Green (CTG) (30 min, 5 μM, 37 °C; ThermoFisher®) in pre-warmed serum-free DMEM and washed in fresh serum-free DMEM during 30 min at 37 °C. Twenty-five thousand cells were seeded in the upper wells of the chemotactic chambers. The lower chamber was filed by 30 μl of serum-free DMEM supplemented with 500 nM CXCL12 (Peprotech®) or control solution. After 16-h incubation in a 5% CO_2_ humidified incubator at 37 °C, cells having migrated in the lower chambers were fixed with 4% paraformaldehyde (PFA) for 15 min and rinsed 3 times with deionized water. The percentage of migrating cells was quantified by counting the number of CTG-positive cells per area by fluorescent microscopy using the Image J software.

### Statistical analyses

Quantitative data are expressed as mean ± SD/SEM of a minimum of three biologically-independent experiments and analyzed by GraphPad Prism 5®. Two-way ANOVA test and Bonferroni post-tests or *t*-tests (two-sided tests) were used to analyze a minimum of three biologically-independent experiments (*n* = 3) and a *p* value of <0.05 was considered as statistically significant. Cell culture, intra-striatal graft, immunofluorescence, Western-blot, quantitative RT-PCR, survival, and migration experiments were previously designed, performed, and validated in the laboratory [7, 9, 27]. Immuno-staining and Western-blot figures show a representative experiment, but all quantitative graphs show the results from the pooling of a minimum of three biologically-independent experiments. Statistical analyses (including ANOVA and *t*-tests) of all sample groups were performed and corrected by Welch and Bonferroni post-tests if appropriate. The sample size was determined by two-tail *t*-tests using the G-Power software. For every figure, statistical tests were justified as appropriate.

## Electronic supplementary material

Supplemental data

Supplemental Figures
