# Supplementary material for: Aurora A plays a dual role in migration and survival of human glioblastoma cells according to the CXCL12 concentration
Source: Oncogene. 2018 Aug 6;38(1):73–87. doi: 10.1038/s41388-018-0437-3 (PMC6755987; doi:10.1038/s41388-018-0437-3)
Supplement: Supplementary file 1 — Supplemental data [file 41388_2018_437_MOESM1_ESM.docx]

**Supplementary Figure Legends**

**Suppl. Fig 1. Phosphoproteome analysis of U87MG by mass spectrometry in U87MG cells after 1h of CXCL12 stimulation** **(A)** The graph shows the main signaling pathways (i.e. serine/threonine kinases, phosphatases, adhesion and extracellular matrix (ECM) proteins and transcriptional/ translational factors) affected by 1hour of CXCL12 stimulation (12.5nM) according to the number of phosphorylated peptides (significant fold change: ±2.5). **(B)** The table shows the fold change of phosphorylation for each residue of the Aurora kinases (AurA, AurB, AurC) and ERK1/2 members in U87MG cells after 1h of CXCL12 stimulation (12.5nM). The residue Thr288 of AurA was significantly dephosphorylation (fold change: -12.4) after 1h of CXCL12 stimulation (12.5nM). Phosphorylation of AurB and AurC were not significantly affected by CXCL12. Residues 198 (fold change” 3.0), 198 and 204 (fold change: 4.8) and 202 (fold change: 2.8) of ERK1 were significantly phosphorylated after 1h of CXCL12 stimulation (12.5nM). Phosphorylation of ERK2 was not significantly affected by CXCL12.

**Suppl. Fig 2.** **Clinical databases analysis of human GBM patients.** Rembrandt data for **(A)** CXCR4, **(B)** Aurka, and **(C)** Ajuba mRNA expression in arbitrary units in GBM (n=214), astrocytomas (n=145), oligodendrogliomas (n=66) and non-cancerous brain tissues (n=21). Non-cancerous tissues include both temporal lobe extracts from epileptic patients and uninvolved brain extracts from cancer patients **(D)** Survival curves of GBM patients treated only by radiotherapy and with high (grey) or low (black) AurkA mRNA expression levels (data extracted from the TGCA database). Survival differences between expression groups were assessed by Kaplan-Meier curves and log-rank tests (“survival” package in R) **(E)** Immunofluorescent staining of CXCR4 (red), CD133 (green) and Hoechst (blue) in GBM1 cells (40X) Scale bar = 10μm.

**Suppl. Fig 3. Dose-response curve of Alisertib and CXCL12 treatments in Boyden’s chambers migration assays.** Boyden’s chambers migration assays on U87MG cells 48 hours after ALS treatment (0nM, 5nM, 10nM, 25nM) in response to CXCL12 (0nM, 250nM, 500nM, 1000nM). Graphs are mean values ±SD and are representative of 3 independent experiments, * p<0.05, ** p<0.01, *** p<0.001

**Suppl. Fig 4. Western-blot analyzes of Rho-GTPase upstream kinases.** Western-blot analyzes of FAK/Tubulin and PKC/Actin in U87MG cells after CXCL12 stimulation (16h, 12.5nM) and Alisertib treatment (48h, 5nM) in U87MG cells.

**Suppl. Fig 5. Characterization of U87MG TM and U87MG SVZ cells** (A) Quantification of spheroids of U87MG TM and U87MG SVZ cells (n=3). Graphs are mean values ±SD and are representative of 3 independent experiments, * p<0.05, ** p<0.01, *** p<0.001 (t test). (B) Immunofluorescent staining of P-AurA (red), Sox2 (green) and Hoechst (blue) in U87MG cells extracted from the tumor mass (TM) or the subventricular zone (SVZ) of GBM-xenografted-mice. (40X). Scale bar=10μM.
