# Supplementary figures and images for: Aurora A plays a dual role in migration and survival of human glioblastoma cells according to the CXCL12 concentration
Source: Oncogene. 2018 Aug 6;38(1):73–87. doi: 10.1038/s41388-018-0437-3 (PMC6755987; doi:10.1038/s41388-018-0437-3)

## Slide 1
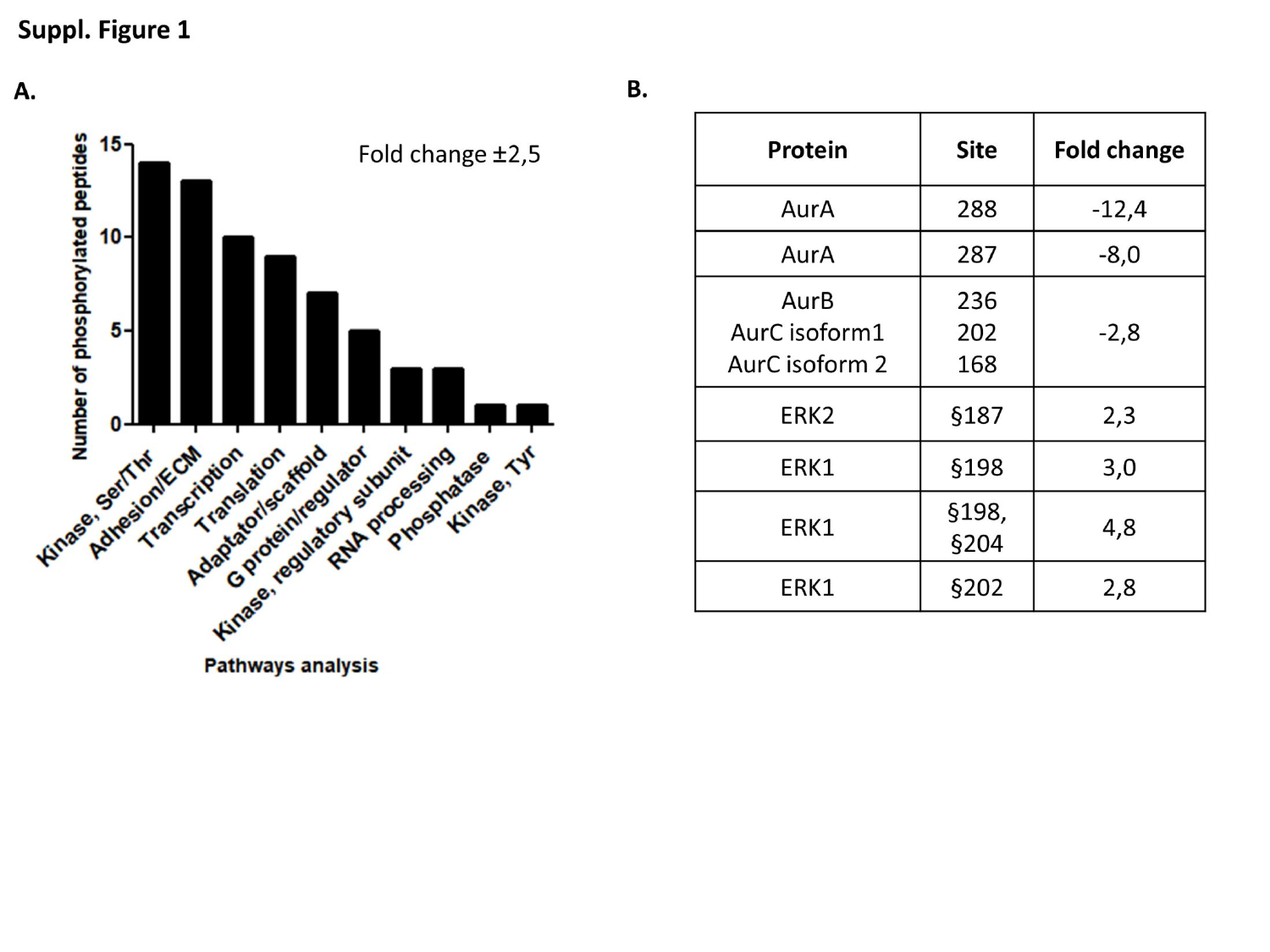

## Slide 2
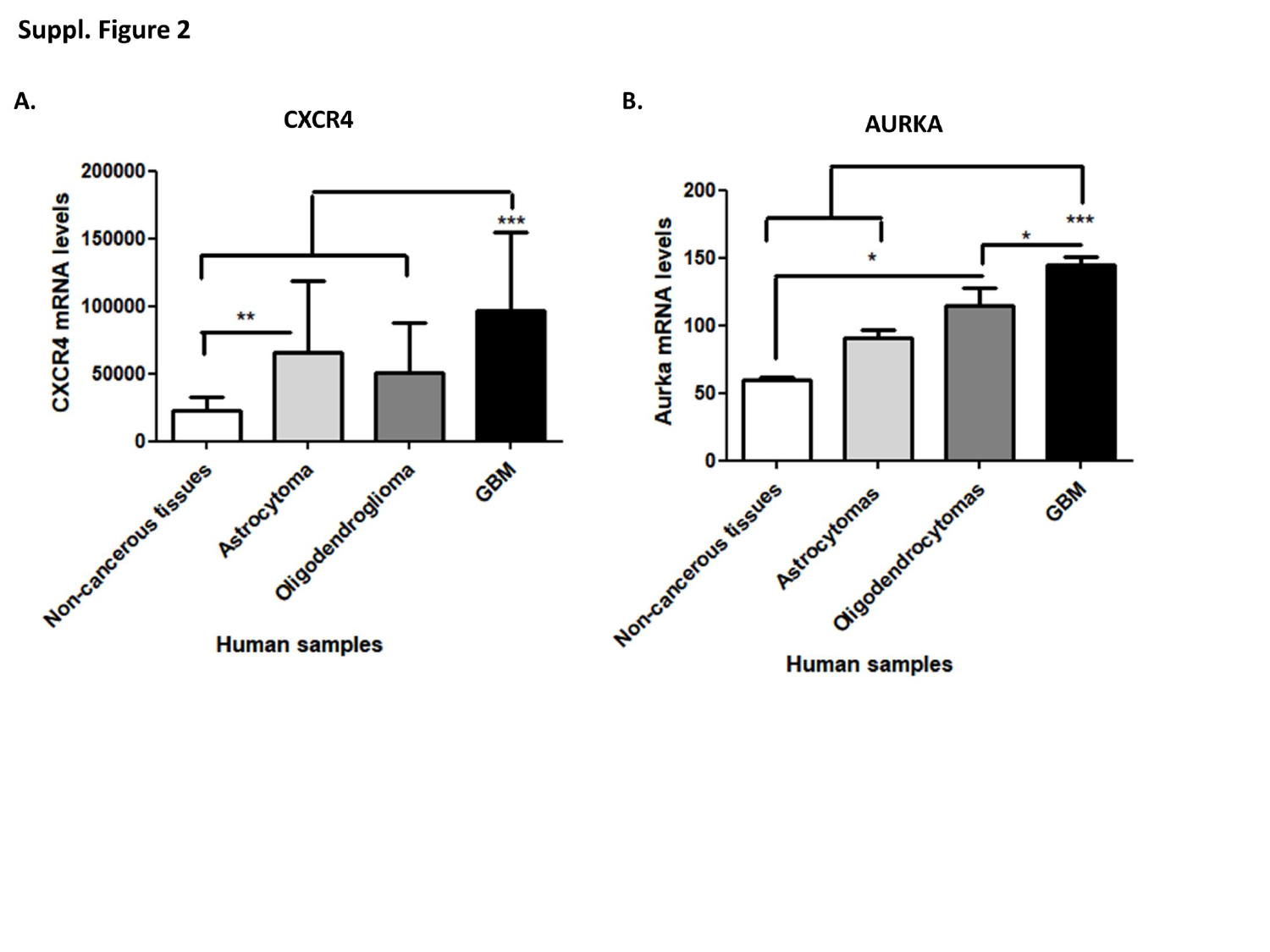

## Slide 3
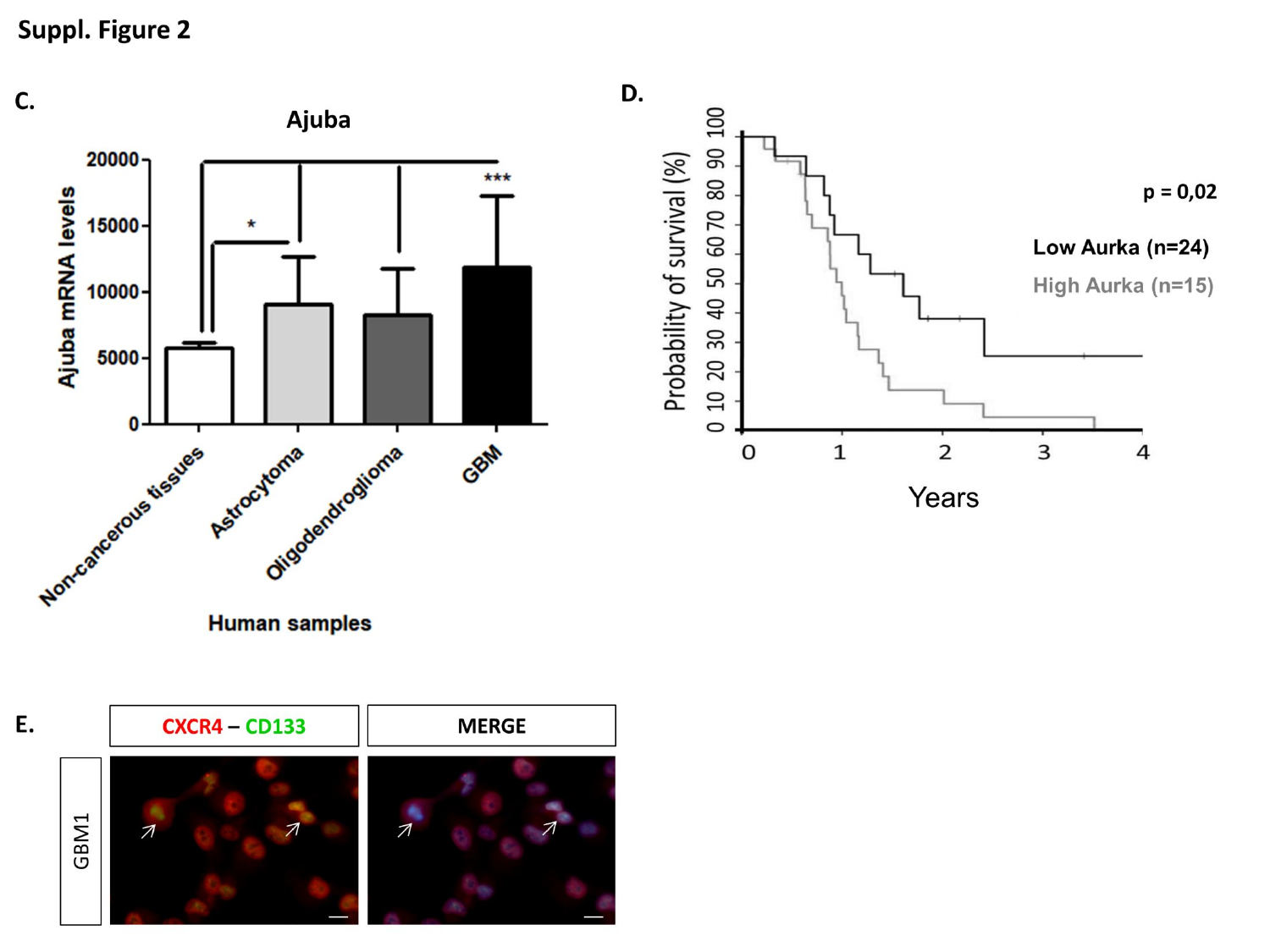

## Slide 4
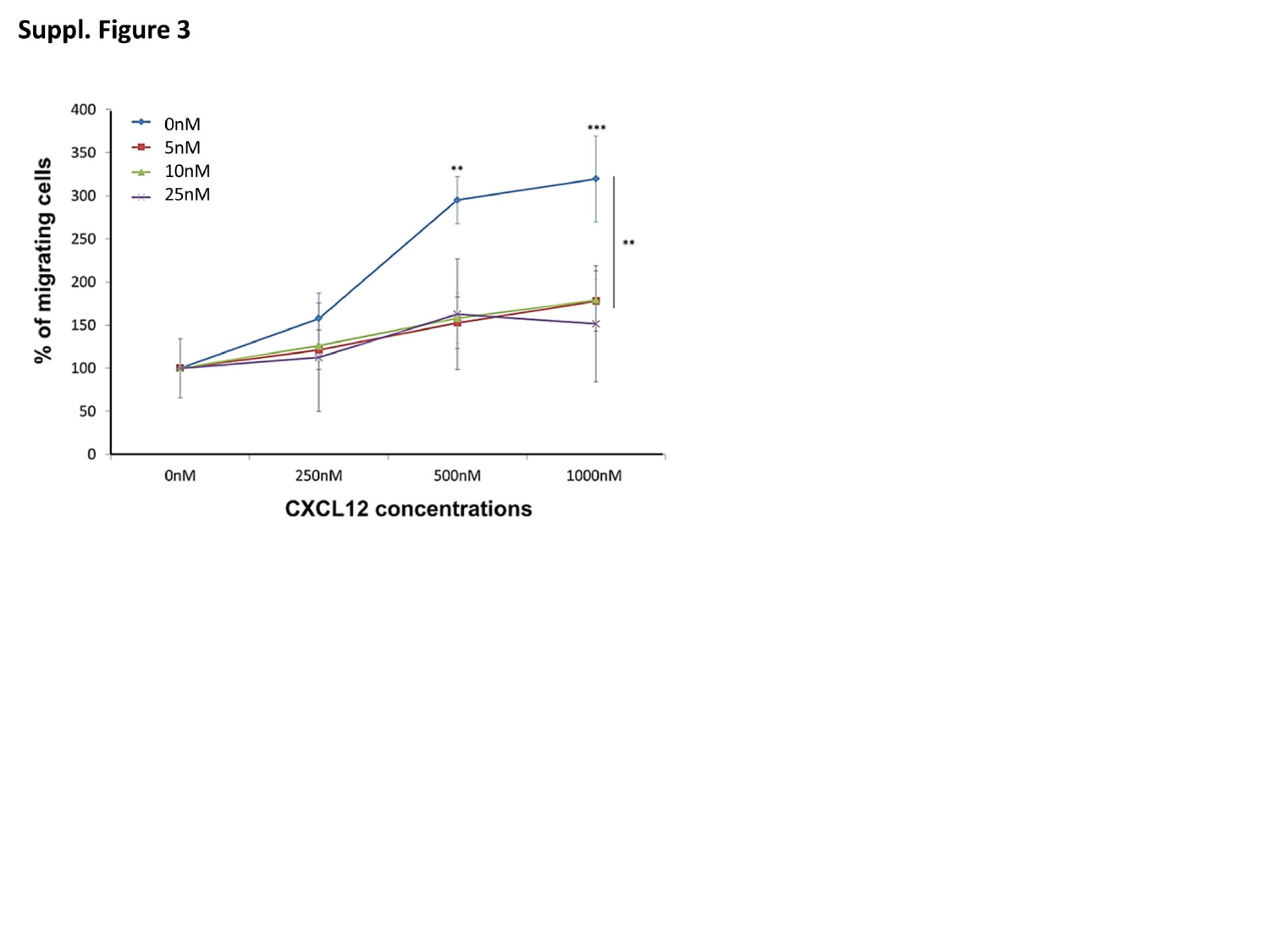

## Slide 5
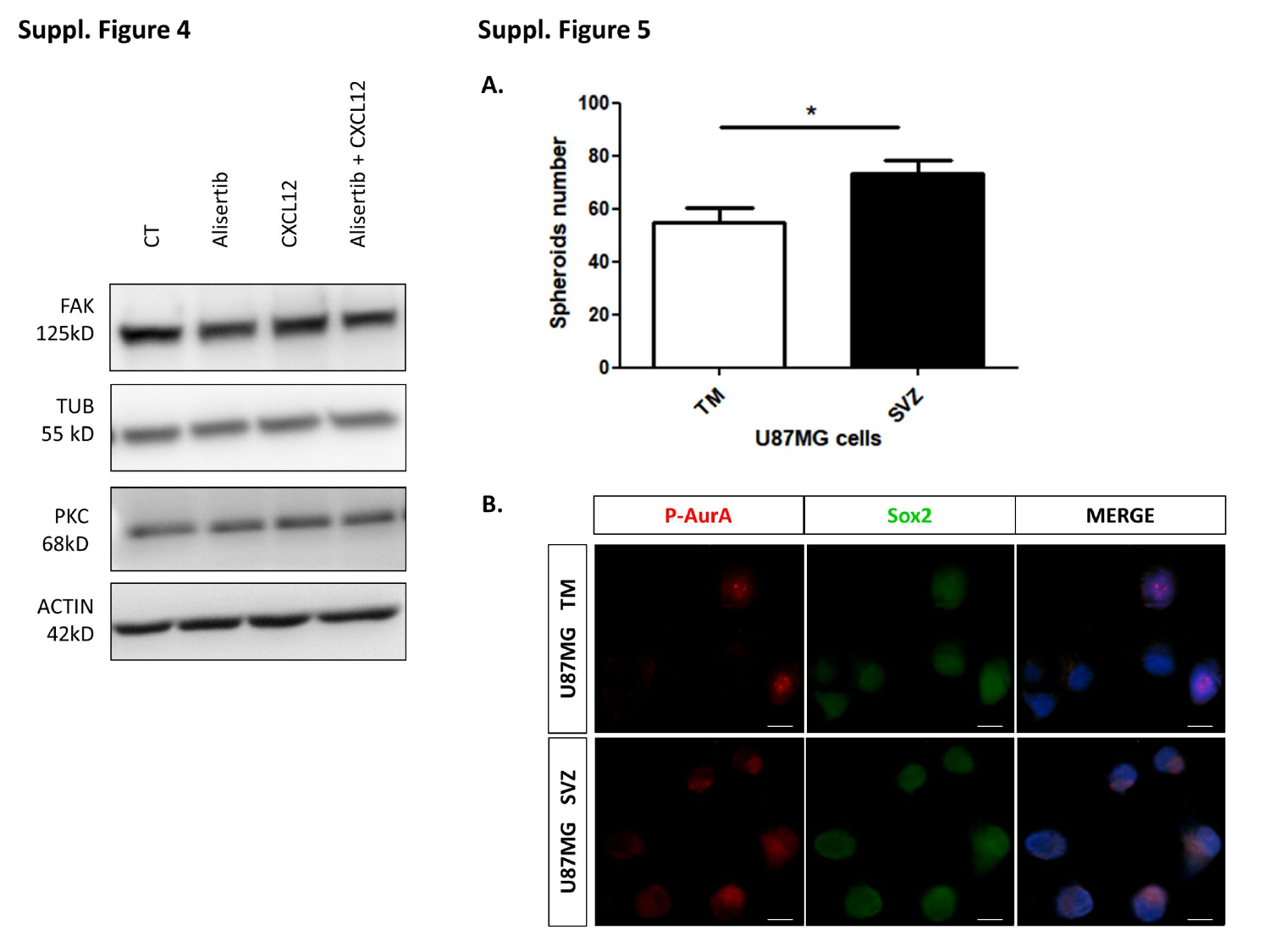

Supplement: Supplementary file 2 — Supplemental Figures [file 41388_2018_437_MOESM2_ESM.pptx]
